# Trends in awareness and uptake of genetic testing among the United States population, 2011–2022

**DOI:** 10.1007/s12687-026-00924-5

**Published:** 2026-08-10

**Authors:** Ariana Naaseh, Mengyao Shi, Steven Tohmasi, Erin L. Linnenbringer, Julie A. Margenthaler, Graham A. Colditz, Yin Cao

**Affiliations:** 1 Division of Public Health Sciences, Department of Surgery, Washington University School of Medicine, Campus Box 8100, 660 S. Euclid Ave., St. Louis, MO 63110 USA; 2 Alvin J. Siteman Cancer Center, Washington University School of Medicine, St. Louis, MO USA; 3 Division of Gastroenterology, Department of Medicine, Washington University School of Medicine, St. Louis, MO USA; 4 Section of Surgical Oncology, Department of Surgery, Washington University School of Medicine, St. Louis, MO USA

## Abstract

While genetic testing (GT) has become more available over the past decade, it is unclear whether public awareness and use have increased proportionally. We aimed to evaluate national trends in GT awareness and usage. We performed a cross-sectional analysis of nationally representative data from the National Cancer Institute’s Health Information National Trends Survey (HINTS). We analyzed survey responses from HINTS 4 Cycle 1–4 (2011–2014), 5 Cycle 1 and 4 (2017, 2020), and 6 (2022). Primary outcomes were overall genetic testing awareness and uptake. Secondary analyses evaluated awareness and uptake of health-related genetic testing (e.g. disease risk, cancer, and carrier testing) among survey cycles in which specific testing modalities were available. Linear regression assessed crude trends and multivariable logistic regression estimated odds ratios (OR). Among 22,256 respondents, overall awareness of GT increased from 36.6% in 2011 to 81.7% in 2022 (trend *p* < .001). Awareness was lower among individuals who were older (e.g.,* ref: age 18–34*,* age 75+: OR 0.53*,* 95% confidence interval [CI]: 0.43–0.64*), male (*ref: female*,* OR 0.85*,* CI: 0.77–0.95*), non-Hispanic Black (*ref: non-Hispanic White*,* OR 0.63*,* CI: 0.54–0.74*), non-Hispanic Asian (*ref: non-Hispanic White*,* OR 0.38*,* CI: 0.29–0.48*), Hispanic (*ref: non-Hispanic White*,* OR 0.60*,* CI: 0.52–0.69*), with less than a college education (e.g.,* ref: some college*,* less than high school: OR 0.60*,* CI: 0.47–0.77*), and with annual household income <$75,000 (e.g.,* ref: >$75*,*000*,* <$20*,*000: OR 0.49*,* CI: 0.42–0.59*). GT uptake increased from 21.4% in 2020 to 35.6% in 2022 (trend *p* < .001). GT uptake was lower among individuals who were non-Hispanic Asian (*ref: non-Hispanic White*,* OR 0.60*,* CI: 0.38–0.96*) and had household incomes <$35,000 (e.g.,* ref: >$75*,*000*,* <$20*,*000: OR 0.68*,* CI: 0.49–0.94*), but higher among those with a personal history of cancer (*ref: no cancer history*,* OR 1.44*,* CI: 1.07–1.92*). Although GT awareness has grown, there are notable gaps across sociodemographic groups. Further study should better characterize factors influencing health-related GT uptake patterns.

**Supplementary Information:**

The online version contains supplementary material available at https://doi.org/10.1007/s12687-026-00924-5.

## Introduction

Genetic testing can be used to assess the risk and tailor prevention or treatment for several disease conditions such as cancer, cardiovascular disease, or hereditary disease. Additionally, genetic testing can be used outside of clinical settings for ancestry or personal trait testing. An emphasis on genetic testing has grown in recent years as new technologies have become available and the medical field continues to advance toward precision medicine. From 2012 to 2022, there has been an increase in the number and type of tests made available throughout the United States (US) (Halbisen and Lu 2023). 

Improving public awareness of genetic testing encompasses increasing knowledge of its availability, clinical indications, potential benefits, and appropriate access pathways. Greater awareness may facilitate informed health-related decision-making, increase appropriate utilization of genetic testing among eligible individuals, and enable earlier identification of inherited disease risk or genetically actionable conditions that can guide prevention, screening, and treatment strategies. However, disparities in awareness may contribute to inequities in access to these potential benefits across sociodemographic groups. Therefore, characterizing national trends in genetic testing awareness and utilization as well as identifying populations with persistently lower awareness are essential to inform targeted educational and public health interventions.

While prior studies have identified subgroup populations with low awareness and uptake of clinically indicated genetic testing, to date, there has not yet been a comprehensive temporal trends analysis of the awareness and uptake of overall genetic testing among the US population over recent years (Canedo et al. 2019, Carroll et al. 2020, Salloum et al. 2018, Williams et al. 2019, Krakow et al. 2017, Tiner et al. 2022). Furthermore, the sociodemographic factors that may influence both behaviors remain poorly understood, despite having the potential to guide future policy changes that may bring our society closer to achieving genetic fairness (e.g., equitable awareness and access to clinically indicated genetic testing across sociodemographic groups). To address this knowledge gap, this study aimed to examine trends in genetic testing awareness and uptake among the US population using national data from the National Cancer Institute’s (NCI) Health Information National Trends Survey (HINTS) from 2011 to 2022.

## Methods

### Study population

This study is a cross-sectional analysis of the HINTS, a nationally representative survey of adults aged 18 years or older in the US performed by the NCI that is designed to assess public awareness and usage of health-related information (Learn More About HINTS | HINTS and Accessed 2024). All non-institutionalized civilian adults are eligible to participate. Details of the HINTS survey have been previously published in detail (Finney Rutten et al. 2012). HINTS surveys have been collected since 2003 and administered every few years, with HINTS 4 and 5 being the only cycles that included annual data collection cycles over the course of three years. All survey cycles that contained genetic testing questions were included in this analysis. For this reason, HINTS 5 Cycle 2 and Cycle 3 were excluded from the analysis. We included survey responses from HINTS 4 Cycle 1 (2011), 4 Cycle 2 (2012), 4 Cycle 3 (2013), 4 Cycle 4 (2014), 5 Cycle 1 (2017), 5 Cycle 4 (2020), and 6 (2022) for analysis in genetic testing awareness and included survey responses from HINTS 5 Cycle 4 (2020) and 6 (2022) for analysis in genetic testing uptake (Figure S1). Our primary analyses evaluated overall genetic testing awareness and uptake across all available genetic testing modalities captured in each survey cycle. Because HINTS 5 Cycle 4 (2020) and HINTS 6 additionally distinguished specific genetic testing modalities, these survey cycles enabled secondary analyses focused on health-related genetic testing awareness and uptake.

### Assessment of genetic testing awareness

Awareness of genetic testing was assessed based on responses to the question “Genetic tests that analyze your DNA, diet, and lifestyle for potential health risks are currently being marketed. Have you heard or read about these genetic tests?” present in HINTS 4 Cycle 1, 4 Cycle 2, and 4 Cycle 4. In HINTS 4 Cycle 3, participants were asked, “Genetic tests are currently being marketed to consumers. Have you heard or read about these genetic tests?” In HINTS 5 Cycle 1, participants were asked “Have you heard or read about this type of genetic test?” All the questions had predefined answers of “Yes” or “No”, which were analyzed as binary variables. In HINTS 5 Cycle 4 and HINTS 6, participants were asked “Which of the following types of genetic tests have you heard of?” In HINTS 5 Cycle 4, predefined response options included: “Ancestry testing”, “Genetic health risk testing”, “High risk cancer testing”, “Other”, “Not sure”, and “I have not heard of any of these tests”. Each of these answer choices was made into a separate binary variable for the purposes of this analysis. Overall genetic testing awareness was defined as awareness of any genetic testing modality included in the respective HINTS survey cycle. For our overall awareness analysis, participants who selected “Ancestry testing”, “Genetic health risk testing”, “High risk cancer testing”, “Other”, or multiple options were coded as being aware of genetic tests. Participants who selected “Not sure” or “I have not heard of any of these tests” were coded as being unaware of genetic tests. In HINTS 6, predefined response options included: “Ancestry testing”, “Personal trait testing”, “Specific disease testing”, “Prenatal genetic carrier testing”, “None of the above”, “Not sure”, and “Other”. Each answer included an explanation of the specific type of genetic test. For example, information about ancestry testing included “to understand where you and your relatives come from, for example tests offered by companies such as Ancestry (Ancestry.com LLC, Lehi, UT) or 23andMe (23andMe Holding Co., San Francisco, CA).” Each answer choice was made into a separate binary variable for the purpose of this analysis. Participants who selected “Ancestry testing”, “Personal trait testing”, “Specific disease testing”, “Prenatal genetic carrier testing”, “Other”, or multiple options were coded as being aware of genetic tests. Participants who selected “None of the above” or “Not sure” were coded as being unaware of genetic tests. Because ancestry and personal trait testing reflect different public health questions than clinically relevant genetic testing, we conducted a prespecified secondary analysis of health-related genetic testing awareness. For this analysis, we further categorized “Genetic health risk testing” and “High risk cancer testing” in HINTS 5 Cycle 4 and “Specific disease testing and “Prenatal genetic carrier testing” in HINTS 6 as health-related genetic testing.

### Assessment of genetic testing uptake

Overall genetic testing uptake was defined as self-reported receipt of any genetic testing modality available within the survey cycle. Participants who selected that they had heard of genetic tests were then offered the question “Which of the following types of genetic tests have you had?” which was used to assess uptake of genetic testing. Of note, this question was only available in HINTS 5 Cycle 4 and HINTS 6. These answer choices included the same options as the questions above in the respective surveys and were recorded into binary variables then coded into a categorical variable in the same fashion to indicate uptake of genetic test or no uptake of genetic test for the overall analysis. We additionally performed a prespecified secondary analysis restricted to health-related genetic testing uptake. Participants who selected “Genetic health risk testing” and “High risk cancer testing” in HINTS 5 Cycle 4 and “Specific disease testing” and “Prenatal genetic carrier testing” in HINTS 6 were categorized as having selected health-related genetic testing.

### Assessment of sociodemographic characteristics

Sociodemographic variables collected included self-reported age, gender, race/ethnicity, education level, and annual household income. Race/ethnicity categories reported in the manuscript were based on those provided in the original survey instrument. Participants were able to self-identify by selecting the category that best reflected their identity, with an “Other” option for those who did not see themselves represented in the listed categories. Personal and family history of cancer were also assessed when available (only present in HINTS 5 Cycle 4 and 6). Each answer selection was recoded as a binary variable then made into a separate categorical variable. Answers of “Yes” were indicative of a person having a family or personal history of cancer and answers of “Not sure” or “No” represented having no family or personal history of cancer.

### Statistical analyses

All analyses used survey weighting procedures with jackknife replicate weights to account for the complex survey design to ensure nationally representative estimates. Estimates on crude weighted prevalence and 95% confidence intervals (CIs) of genetic testing awareness and uptake were calculated by cycle. Crude linear trend in genetic testing awareness and uptake was evaluated using a linear regression model across survey cycles and to estimate regression coefficients (β) and CIs for annual change. P for trend was estimated using the survey cycle as a continuous variable. Additionally, the proportion of different genetic testing types by genetic testing awareness and uptake for the most recent cycle were visually illustrated.

Multivariable logistic regression was used to estimate odds ratios (ORs) and CIs of overall genetic testing awareness and uptake, adjusting for age (18–34, 35–49, 50–64, 65–74, 75+), gender (male, female), race/ethnicity (non-Hispanic White, non-Hispanic Black, non-Hispanic Asian, non-Hispanic Other, Hispanic), education level (less than high school, 12 years or completed high school, some college, college graduate or more), annual household income (<$20,000, $20,000 to <$35,000, $35,000 to <$50,000, $50,000 to <$75,000, $75,000 or more), personal history of cancer (yes, no), family history of cancer (yes, no), and cycle (HINTS 4 Cycle 1, HINTS 4 Cycle 2, HINTS 4 Cycle 3, HINTS 4 Cycle 4, HINTS 5 Cycle 1, HINTS 5 Cycle 4, HINTS 6). The primary analyses evaluated overall genetic testing awareness and uptake because these outcomes were consistently measurable across the available HINTS survey cycles and reflect population-level trends in familiarity and use of genetic testing. To better characterize clinically relevant testing behaviors, we conducted prespecified secondary analyses restricted to health-related genetic testing awareness and uptake among participants in HINTS 5 Cycle 4 and HINTS 6, the only survey cycles that distinguished testing modality. These secondary analyses allowed us to evaluate whether sociodemographic patterns differed for clinically oriented genetic testing compared with overall genetic testing. All analyses were performed using R (version 4.2.3) and considered significant with two-sided *P* < .05.

## Results

**Fig. 1:**
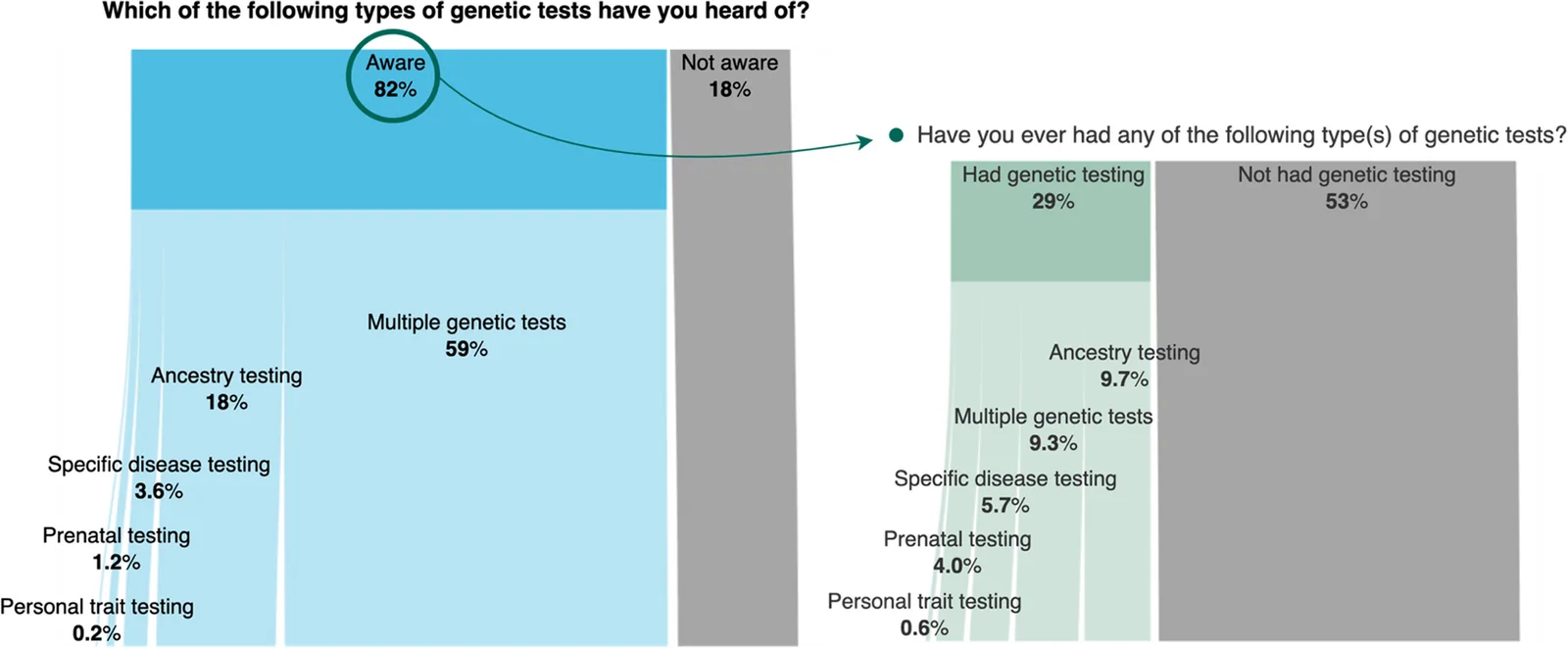
Proportion of different genetic testing types for genetic testing awareness and genetic testing uptake, HINTS 2022

Unweighted sample sizes and weighted percentages for all cycles by sociodemographic characteristics are presented in Table 1. The proportion of different genetic testing types by genetic testing awareness and uptake for HINTS Cycle 6 are displayed in Fig. 1. Weighted sample sizes by sociodemographic characteristics in HINTS Cycle 6 and across all cycles are presented in Table S1 and Table S2. In the HINTS 6 cohort of 5186 respondents, the mean (SD) age at baseline was 48.4 (17.5) years, 3101 participants (50.7%) were women, and 2978 participants (61.5%) were non-Hispanic White.

**Table 1:** Sample Size for Genetic Testing Awareness and Uptake in the US Population by Sociodemographic Characteristics, HINTS 2011–2022

Characteristics	HINTS 4 Cycle 1	HINTS 4 Cycle 2	HINTS 4 Cycle 3	HINTS 4 Cycle 4	HINTS 5 Cycle 1	HINTS 5 Cycle 4	HINTS 6	Total
Year	2011	2012	2013	2014	2017	2020	2022	2011–2022
No. of participants	3271	2806	2373	2875	2641	3104	5186	22,256
Age, mean (SD)	45.3 (17.4)	44.6 (17.1)	45.4 (16.8)	47.6 (16.0)	48.0 (16.4)	47.0 (17.5)	48.4 (17.5)	46.7 (17.0)
18–34	541 (32.0)	486 (33.5)	374 (30.3)	434 (24.2)	345 (22.9)	453 (28.0)	792 (25.6)	3425 (28.0)
35–49	838 (28.1)	741 (28.1)	595 (30.8)	671 (28.5)	601 (30.6)	651 (27.3)	1088 (26.4)	5185 (28.5)
50–64	1138 (25.5)	951 (25.2)	841 (24.6)	1021 (32.4)	915 (30.5)	948 (27.0)	1524 (28.0)	7338 (27.6)
65–74	443 (8.1)	386 (7.8)	362 (8.7)	470 (8.9)	518 (9.5)	701 (11.1)	1138 (12.6)	4018 (9.6)
75+	311 (6.3)	242 (5.4)	201 (5.7)	279 (5.9)	262 (6.5)	351 (6.6)	644 (7.6)	2290 (6.3)
Sex								
Male	1345 (49.3)	1105 (49.5)	950 (49.6)	1134 (48.4)	1099 (49.4)	1330 (49.6)	2085 (49.3)	9048 (49.3)
Female	1926 (50.6)	1701 (50.5)	1423 (50.4)	1741 (51.6)	1542 (50.6)	1774 (50.4)	3101 (50.7)	13,208 (50.7)
Race/ethnicity								
Non-Hispanic White	2129 (67.0)	1770 (67.3)	1394 (66.7)	1710 (65.4)	1671 (65.4)	1907 (63.7)	2978 (61.5)	13,559 (65.2)
Non-Hispanic Black	491 (11.1)	408 (10.4)	350 (9.7)	476 (11.1)	367 (10.4)	406 (10.9)	831 (11.1)	3329 (10.7)
Non-Hispanic Asian	153 (5.4)	90 (4.9)	100 (5.2)	115 (5.3)	129 (5.8)	148 (5.2)	273 (6.0)	1008 (5.4)
Non-Hispanic Other	90 (2.2)	95 (2.3)	90 (2.9)	102 (2.6)	97 (2.6)	111 (3.3)	176 (4.6)	761 (3.0)
Hispanic	408 (14.4)	443 (15.0)	439 (15.4)	472 (15.5)	377 (15.8)	532 (17.0)	928 (16.8)	3599 (15.7)
Education level								
Less than HS	298 (11.7)	215 (11.1)	191 (7.8)	226 (8.4)	154 (8.0)	199 (7.4)	313 (6.4)	1596 (8.6)
12 years or completed HS	622 (21.2)	544 (18.7)	482 (21.8)	505 (22.0)	442 (20.8)	523 (20.8)	891 (20.8)	4009 (20.9)
Some college	996 (32.6)	872 (40.0)	726 (34.5)	891 (33.1)	772 (32.5)	922 (40.3)	1477 (39.2)	6656 (36.1)
College graduate or higher	1355 (34.5)	1175 (30.1)	974 (35.9)	1253 (36.4)	1273 (38.7)	1460 (31.5)	2505 (33.7)	9995 (34.4)
Annual household income								
< 20k	725 (22.8)	603 (20.3)	541 (18.8)	624 (18.1)	440 (15.6)	495 (13.9)	840 (13.7)	4268 (17.5)
20-<35k	531 (17.4)	438 (14.3)	348 (13.9)	414 (11.8)	368 (12.2)	399 (11.2)	672 (10.8)	3170 (13.0)
35-<50	480 (12.5)	407 (15.8)	340 (14.8)	423 (15.7)	332 (14.3)	408 (12.1)	683 (11.8)	3073 (13.8)
50-<75k	557 (17.3)	489 (17.5)	399 (17.9)	501 (17.9)	492 (19.6)	552 (18.9)	898 (18.6)	3888 (18.3)
75k and more	978 (29.9)	869 (32.0)	745 (34.6)	913 (36.4)	1009 (38.3)	1250 (43.9)	2093 (45.2)	7857 (37.4)
Personal history of cancer^b^								
Yes	-	-	-	-	-	481 (8.4)	763 (9.6)	1244 (9.0)
No	-	-	-	-	-	2621 (91.6)	4421 (90.4)	7042 (91.0)
Family history of cancer^b^								
Yes	-	-	-	-	-	2236 (71.2)	3558 (65.0)	5794 (68.1)
No	-	-	-	-	-	835 (28.8)	1601 (35.0)	2436 (31.9)

*Abbreviations*: *HINTS* Health Information Trends Survey, *HS* High school, *SD* Standard deviation

^a^Data was presented as unweighted number (weighted %)

^b^Information on personal history of cancer and family history of cancer was only collected in HINTS 5 Cycle 4 and HINTS 6

### Genetic testing awareness

Crude weighted percentages of genetic testing awareness by cycle are presented in Table 2. The prevalence of genetic testing awareness increased by 4.3% per year, increasing from 36.6% in 2011 to 81.7% in 2022 (P for trend < 0.001).

**Table 2:** Crude weighted trends in genetic testing awareness and uptake among the US population, HINTS 2011–2022

HINTS cycle years							β (95% CI)^b^	*P* for trend^b^
2011	2012	2013	2014	2017	2020	2022		
Genetic testing awareness								
Weighted % (95% CI)^a^								
36.6 (32.4 to 39.0)	51.0 (48.1 to 53.8)	39.3 (36.5 to 42.3)	37. 9 (35.3 to 40.6)	59.3 (56.9 to 61.6)	78.8 (75.8 to 81.5)	81.7 (79.9 to 83.4)	4.3 (4.0 to 4.5)	*P*<.001
OR (95% CI)^c^								
1 [Reference]	1.86 (1.58–2.18)	1.07 (0.90–1.26)	1.01 (0.86–1.18)	2.53 (2.17–2.94)	6.91 (5.61–8.50)	8.34 (7.14–9.75)		
Genetic testing uptake								
Weighted % (95% CI)^a^								
-	-	-	-	-	21.4 (19.2 to 23.8)	35.6 (33.1 to 38.3)	7.1 (5.4 to 8.8)	*P*<.001
OR (95% CI)^c^								
-	-	-	-	-	1 [Reference]	2.01 (1.67–2.41)		

^a^Weighted estimates and 95% confidence intervals (CI) were estimated for each survey cycle interchange

^b^The estimate β, 95% CI, and P for trend were calculated using linear regression that included the HINTS cycle as a continuous variable. The estimate β can be interpreted as the average percentage point change in prevalence every year

^c^Adjusted for age (18–34, 35–49, 50–64, 65–74, 75+), gender (male, female), race/ethnicity (non-Hispanic White, non-Hispanic Black, non-Hispanic Asian, non-Hispanic Other, Hispanic), education level (less than high school, 12 years or completed high school, some college, college graduate or more), and annual household income (less than $20,000, $20,000 to <$35,000, $35,000 to <$50,000, $50,000 to <$75,000, $75,000 or more)

After multivariable adjustment, individuals who were older, male, non-Hispanic Black, non-Hispanic Asian, Hispanic, with an education level less than some college, and with annual household income less than $75,000 were significantly less likely to have heard of any type of genetic testing across all cycles (Table 3). Compared to individuals aged 18–34, those aged 35–49 (OR, 0.81; CI, 0.69–0.95) and 75 and older (OR, 0.53; CI, 0.43–0.64) were significantly less likely to be aware of genetic testing. Male individuals were significantly less likely to have heard of genetic testing than females (OR, 0.85; CI, 0.77–0.95). Individuals who were Hispanic (OR, 0.60; CI, 0.52–0.69), non-Hispanic Asian (OR, 0.38; CI, 0.29–0.48), and non-Hispanic Black (OR, 0.63; CI, 0.54–0.74) were significantly less likely to be aware of genetic testing than non-Hispanic White individuals. Compared with those with some college education, individuals with education less than high school (OR, 0.60; CI, 0.47–0.77) and twelve years or completed high school (OR, 0.66; CI, 0.57–0.77) were significantly less likely to have heard of genetic testing. In contrast, those with a college graduate degree or higher were significantly more likely to have heard of genetic testing (OR, 1.51; CI, 1.33–1.71). Compared to individuals with an annual household income of $75,000 or more, individuals with an annual household income of less than $20,000 (OR, 0.49; CI, 0.42–0.59), $20,000 to less than $35,000 (OR, 0.56; CI, 0.48–0.67), $35,000 to less than $50,000 (OR, 0.63; 95% CI, 0.53–0.75), and $50,000 to less than $75,000 (OR, 0.74; CI, 0.65–0.86) were significantly less likely to be aware of genetic testing.

**Table 3:** Weighted logistic regression models of genetic testing awareness overall, HINTS 2011–2022

	Genetic testing awareness	
	Weighted % (95% CI)	OR (95% CI)^a^
Number of participants	22,256	
Age		
18–34	56.3 (53.7 to 58.8)	1 [Reference]
35–49	54.0 (52.0 to 56.0)	0.81 (0.69–0.95)
50–64	59.0 (57.5 to 60.5)	0.98 (0.85–1.13)
65–74	57.3 (55.2 to 59.4)	0.84 (0.70-1.00)
75+	44.5 (41.6 to 47.4)	0.53 (0.43–0.64)
Gender		
Female	57.0 (55.7 to 58.3)	1 [Reference]
Male	54.4 (52.8 to 56.0)	0.85 (0.77–0.95)
Race/ethnicity		
Non-Hispanic White	59.7 (58.5 to 60.9)	1 [Reference]
Non-Hispanic Black	47.8 (44.9 to 50.7)	0.63 (0.54–0.74)
Non-Hispanic Asian	45.4 (40.5 to 50.4)	0.38 (0.29–0.48)
Non-Hispanic Other	62.2 (55.7 to 68.2)	0.97 (0.71–1.31)
Hispanic	47.0 (44.5 to 49.5)	0.60 (0.52–0.69)
Education level		
Less than high school	34.9 (30.9 to 39.1)	0.60 (0.47–0.77)
12 years or completed high school	44.7 (42.2 to 47.2)	0.66 (0.57–0.77)
Some college	57.7 (55.8 to 59.5)	1 [Reference]
College graduate or higher	65.6 (64.3 to 66.9)	1.51 (1.33–1.71)
Annual household income		
< 20k	40.4 (37.6 to 43.3)	0.49 (0.42–0.59)
20-<35k	45.4 (42.4 to 48.3)	0.56 (0.48–0.67)
35-<50k	50. 0 (46.8 to 53.2)	0.63 (0.53–0.75)
50-<75k	57.2 (54.8 to 59.6)	0.74 (0.65–0.86)
75k and more	67.9 (66.3 to 69.4)	1 [Reference]

^a^Adjusted for age (18–34, 35–49, 50–64, 65–74, 75+), gender (male, female), race/ethnicity (non-Hispanic White, non-Hispanic Black, non-Hispanic Asian, non-Hispanic Other, Hispanic), education level (less than high school, 12 years or completed high school, some college, college graduate or more), annual household income (less than $20,000, $20,000 to <$35,000, $35,000 to <$50,000, $50,000 to <$75,000, $75,000 or more), and cycle (HINTS 4 Cycle 1, HINTS 4 Cycle 2, HINTS 4 Cycle 3, HINTS 4 Cycle 4, HINTS 5 Cycle 1, HINTS 5 Cycle 4, HINTS 6)

The results of our health-related genetic testing awareness sub analysis are presented in Table S3. After multivariable adjustment, male individuals were significantly less likely to be aware of health-related genetic tests than females (OR, 0.49; CI, 0.33–0.72).

### Genetic testing uptake

Crude weighted percentages of genetic testing uptake for HINTS 5 Cycle 4 and HINTS 6 are presented in Table 2. Uptake of overall genetic testing increased by 7.1% between the two cycles, from 21.4% in 2020 to 35.6% in 2022 (P for trend < 0.001).

After multivariable adjustment, individuals who were significantly less likely to have had any type of genetic testing were male, non-Hispanic Asian, and had annual household incomes less than $35,000 or between $50,000 to $75,000 (Table S4). When compared to adults aged 18–34, individuals aged 35–49 (OR, 1.45; CI, 1.07–1.96), 50–64 (OR, 1.31; CI, 1.01–1.69), and older than 75 (OR, 1.53; CI, 1.05–2.22) were significantly more likely to have completed genetic testing. Compared to non-Hispanic White individuals, non-Hispanic Asian individuals were significantly less likely to complete genetic testing (OR, 0.60; CI, 0.38–0.96), while non-Hispanic Other individuals were significantly more likely to complete genetic testing (OR, 1.69; CI, 1.04–2.74). Compared to individuals with an annual income of $75,000 or more, individuals with an income less than $20,000 (OR, 0.68; CI, 0.49–0.94), $20,000 to less than $35,000 (OR, 0.66; CI, 0.45–0.96), and $50,000 to $75,000 (OR, 0.67; CI, 0.53–0.84) were significantly less likely to complete genetic testing. Individuals with a personal history of cancer were significantly more likely to complete genetic testing than those without (OR, 1.44; CI, 1.07–1.92).

The results of our health-related genetic testing uptake sub analysis are presented in Table 4. After multivariable adjustment, individuals aged 35–49 were significantly more likely to have completed health-related genetic testing than those aged 18–34 (OR, 1.83; CI, 1.06–3.15). Male individuals were significantly less likely to have completed health-related genetic testing than females (OR, 0.43; CI, 0.31–0.61). Individuals with a personal history of cancer were significantly more likely to complete health-related genetic testing than those without (OR, 2.88; CI, 1.79–4.62).

**Table 4:** Weighted logistic regression models of health-related genetic testing uptake, HINTS 2020–2022

	Health-related genetic testing uptake	
	Weighted %	OR (95% CI)^a^
Number of participants	4819	
Age		
18–34	8.0 (5.3 to 11.8)	1 [Reference]
35–49	14.7 (11.7 to 18.3)	1.83 (1.06–3.15)
50–64	10.2 (8.3 to 12.5)	1.04 (0.63–1.72)
65–74	9.3 (7.3 to 11.8)	0.71 (0.36–1.42)
75+	8.8 (5.0 to 14.8)	0.60 (0.27–1.36)
Gender		
Female	14.5 (12.7 to 16.4)	1 [Reference]
Male	6.7 (4.7 to 9.3)	0.43 (0.31–0.61)
Race/ethnicity		
Non-Hispanic White	10.7 (8.9 to 12.9)	1 [Reference]
Non-Hispanic Black	12.5 (9.1 to 16.9)	1.22 (0.79–1.89)
Non-Hispanic Asian	8.1 (4.9 to 13.0)	0.76 (0.40–1.45)
Non-Hispanic Other	17.7 (8.6 to 32.9)	1.68 (0.79–3.60)
Hispanic	7.9 (5.7 to 10.8)	0.77 (0.45–1.31)
Education level		
Less than high school	14.0 (5.7 to 30.6)	1.95 (0.59–6.43)
12 years or completed high school	11.4 (7.2 to 17.6)	1.35 (0.74–2.48)
Some college	9.0 (6.9 to 11.6)	1 [Reference]
College graduate or higher	11.5 (9.9 to 13.2)	1.22 (0.84–1.77)
Annual household income		
< 20k	12.2 (8.3 to 17.7)	1.06 (0.64–1.76)
20-<35k	11.5 (6.9 to 18.7)	1.04 (0.56–1.96)
35-<50	13.2 (7.6 to 21.8)	1.34 (0.71–2.50)
50-<75k	7.7 (5.6 to 10.4)	0.70 (0.45–1.08)
75k and more	10.6 (9.0 to 12.4)	1 [Reference]
Personal history of cancer		
No	9.6 (8.1 to 11.5)	1 [Reference]
Yes	21.0 (16.1 to 26.8)	2.88 (1.79–4.62)
Family history of cancer		
No	8.5 (5.9 to 12.0)	1 [Reference]
Yes	11.5 (9.7 to 13.5)	1.31 (0.86–2.00)

^a^Adjusted for age (18–34, 35–49, 50–64, 65–74, 75+), gender (male, female), race/ethnicity (non-Hispanic White, non-Hispanic Black, non-Hispanic Asian, non-Hispanic Other, Hispanic), education level (less than high school, 12 years or completed high school, some college, college graduate or more), annual household income (less than $20,000, $20,000 to <$35,000, $35,000 to <$50,000, $50,000 to <$75,000, $75,000 or more), personal history of cancer (yes, no), family history of cancer (yes, no), and cycle (HINTS 5 Cycle 4, HINTS 6)

## Discussion

Leveraging the results from a validated, nationally representative survey, our findings indicate that the awareness and uptake of genetic testing has increased among the US population between 2011 and 2022. Notably, the prevalence of overall genetic testing awareness and uptake was 81.7% and 35.6%, respectively, in 2022. The observed increase in genetic testing awareness and utilization over the study period likely reflects several concurrent developments in both clinical practice and direct-to-consumer testing. Advances in sequencing technologies, expanded clinical guidelines and insurance coverage, and growing integration of precision medicine have increased the accessibility and use of medically indicated genetic testing (Bastarache et al. 2025, Bensouna et al. 2025). At the same time, the rapid expansion and widespread marketing of direct-to-consumer ancestry and health-related genetic testing services have likely contributed to increased public awareness and uptake of genetic testing more broadly (O’Daniel et al. 2025). Our findings align with the previously published literature demonstrating that ancestry testing is the most commonly recognized and utilized form of genetic testing within the US population (Fahim et al. 2024). Additionally, the US population continues to demonstrate inequalities in awareness and uptake of genetic testing persisting across racial, educational, socioeconomic, gender, and age groups (Pagán et al. 2009, Wideroff et al. 2003, Hann et al. 2017, Apathy et al. 2018, Kolor et al. 2012, Christensen et al. 2023, Haga et al. 2013, Chin and Tham 2020). Our contemporary cross-sectional trend analysis, spanning multiple HINTS cycles, demonstrates that these gaps, previously documented in non-nationally representative samples and in international settings, have persisted over time. Even in 2022, greater overall genetic testing awareness remains associated with higher education, non-Hispanic White race, female gender, and higher income brackets.

The sociodemographic disparities in genetic testing awareness and uptake observed in our study likely reflect a complex interplay of structural, informational, and socioeconomic factors. Although our cross-sectional analysis cannot determine the underlying causes of these disparities, prior studies provide insight into possible explanations for observed associations. Huang et al. demonstrated that health-related knowledge, online information-seeking behaviors, and information trust of the Internet were correlated with prediction of awareness of online genetic testing information in different racial and ethnic groups (HUANG and APOUEY 2014). Similarly, Agurs-Collins et al. demonstrated that the odds of awareness of direct-to-consumer genetic tests were significantly higher for those who actively sought out cancer information, had internet proficiency, and had higher numeracy skills (Agurs-Collins et al. 2015). Together, these prior studies suggest that differences in individuals’ ability to access and process health information may contribute to disparities in genetic testing awareness and uptake, although these mechanisms were not directly evaluated in our study. To address potential downstream health consequences stemming from these observed disparities, education efforts for all are essential, along with measures to improve access to health-related information for individuals with low health and technology literacy. Further qualitative and implementation science research is needed to narrow and eventually close these gaps identified by characterizing facilitators and barriers to the receipt of genetic testing (Hamilton et al. 2016, Ochieng et al. 2024). Participatory research among community members can also be applied across various groups to assist public health practitioners and policymakers in creating and implementing appropriate interventions to increase genetic testing rates in at-risk communities.

Genetic counseling is generally recommended for individuals with a personal or family history suggestive of an inherited condition, early-onset disease, multiple affected relatives, or other clinical features consistent with a hereditary syndrome. In these settings, consultation with a genetics professional can help determine the appropriateness of genetic testing, facilitate interpretation of results, and guide individualized risk assessment and management. Our study found that although genetic testing awareness and uptake are rising nationally, only a small percentage of the population has completed any form of genetic testing. While we are unable to determine which individuals in our sample had clinical indications for health-related genetic testing, prior research in the context of hereditary cancer syndromes and cascade testing has identified several barriers that may prevent an individual from completing genetic testing and contribute to lower-than-expected uptake among eligible individuals. These reasons can range from individual- to system-level factors. Personal concerns can include limited knowledge of genetics, psychosocial distress such as worry or fear, genetic discrimination, medical distrust, logistics related to healthcare utilization, or religious beliefs (Hanson et al. 2023, Guo et al. 2022, Blomen et al. 2021). Provider concerns can include limited genetics knowledge and risk assessment, patient-provider communication, referral patterns, and limited access to specialty referral providers. Systemic concerns can include challenges with availability of genetic services, especially in low resource settings (Rodriguez et al. 2023). Various interventions have been proposed, primarily at single institutions, to improve uptake of genetic testing such as increased provider education or streamlining genetic testing within one clinical space or pathway with demonstrated short-term success (Kukafka et al. 2022, Marjon et al. 2023). While our data do not allow us to assess these barriers directly or determine whether they explain the associations observed in our study, understanding these known factors remains critical to interpreting persistent gaps in uptake and guiding future research and intervention design.

The study has several strengths. First, it utilizes a nationally representative dataset, ensuring broad generalizability of findings to the US population. The serial, cross-sectional design allows for the examination of temporal trends in genetic testing awareness and uptake over more than a decade, providing valuable contemporary insights into changing patterns. Additionally, the large sample size enhances statistical power and enables robust subgroup analyses across key sociodemographic variables include age, gender, race/ethnicity, education, and income. This study also benefits from its focus on both awareness and uptake of genetic testing, distinguishing the two and highlighting differences in access and utilization. Lastly, by identifying populations with lower awareness and uptake, the study underscores critical areas for interventions and future research.

This study also has some limitations. First, survey questions on uptake were only available to respondents aware of genetic testing. While awareness is associated with greater interest in genetic testing and discussions with healthcare providers, lack of awareness does not preclude uptake and may have influenced our prevalence estimates (Apathy et al. 2018). Second, the cross-sectional, self-reported nature of the HINTS survey precludes causal inference. Consequently, the associations observed between sociodemographic characteristics and genetic testing awareness or uptake should not be interpreted as evidence of causal relationships, and the potential mechanisms discussed are hypotheses informed by prior literature rather than conclusions drawn from our data. Additionally, prior HINTS studies on cancer genetic testing found that individuals with a family history of cancer were more likely to be aware of genetic testing, while those with a personal history of cancer were more likely to undergo testing (Tiner et al. 2022). Our overall trend analysis includes various genetic tests, such as ancestry and health-risk testing, which may explain discrepancies in awareness trends among individuals with a family history of cancer. This could highlight an opportunity to improve care by identifying and counseling at-risk individuals about genetic testing.

Importantly, the HINTS survey instrument evolved over time, particularly with respect to assessment of genetic testing awareness. Earlier survey cycles used broad awareness questions, whereas later cycles included multiple specific testing categories and examples of commercially available tests. These changes may have increased respondents’ recognition of genetic testing and could have contributed to some of the observed increase in awareness independent of true changes in population knowledge. Nevertheless, the consistent upward trend observed over more than a decade likely reflects both increasing public awareness as genetic testing became more widely available and publicized as well as changes in survey design. Accordingly, temporal considerations should be interpreted with appropriate caution. Additional limitations should also be considered. HINTS is administered in only English and Spanish, potentially limiting representation of individuals who speak other languages and introducing variability in the comprehension of genetic testing terminology. In our analyses, respondents who selected “Not sure” were classified as not being aware of genetic testing; however, this response may reflect uncertainty or incomplete knowledge rather than a true lack of awareness. Additionally, household incomes was categorized using nominal income thresholds that were not adjusted for inflation across survey years, which may have influenced temporal comparisons by income category. Despite these limitations, HINTS provides a nationally representative sample from which temporal trends in genetic testing and uptake can be estimated.

## Conclusions

Our cross-sectional trend analyses demonstrate that both genetic testing awareness and uptake have increased in the US since 2011. However, important disparities persist. Genetic testing awareness was lower among individuals who were older, male, non-Hispanic Black, non-Hispanic Asian, Hispanic, had less than a college education, or reported an annual household income below $75,000. Similarly, genetic testing uptake was lower among non-Hispanic Asian individuals and those with annual household incomes below $35,000, while it was higher among individuals with a personal history of cancer. These findings highlight the need for continued investigation into the factors influencing genetic testing awareness and uptake across the U.S. population. Targeted research, outreach, and educational efforts are needed to address these persistent disparities and advance equitable access to genetic testing.

## Supplementary Information


Supplementary Material 1.



Supplementary Material 2.



Supplementary Material 3.



Supplementary Material 4.



Supplementary Material 5.


## Data Availability

The data used in this study are publicly available and can be accessed free of charge from the National Cancer Institute at https://hints.cancer.gov/data/download-data.aspx. No additional data were generated or analyzed beyond those available in the public dataset.
