# Supplementary material for: Trends in awareness and uptake of genetic testing among the United States population, 2011–2022
Source: J Community Genet. 2026 Aug 10;17(4):95. doi: 10.1007/s12687-026-00924-5 (PMC13457883; doi:10.1007/s12687-026-00924-5)
Supplement: Supplementary file 1 [file 12687_2026_924_MOESM1_ESM.docx]

**Table S1**. Sample Size for Genetic Testing Awareness and Uptake in the US Population by Sociodemographic Characteristics, HINTS 2022.

| **Characteristics** | **HINTS 6** |
| --- | --- |
| Year | 2022 |
| **No. of participants** | 10,893,389,976 |
| **Age, mean (SD)** |  |
| 18-34 | 55,744,495 (25.6) |
| 35-49 | 57,409,116 (26.4) |
| 50-64 | 60,869,613 (28.0) |
| 65-74 | 27,378,482 (12.6) |
| 75+ | 16,461,979 (7.6) |
| **Gender** |  |
| Male | 107,510,556 (49.3) |
| Female | 110,353,128 (50.7) |
| **Race/ethnicity** |  |
| Non-Hispanic White | 134,080,124 (61.5) |
| Non-Hispanic Black | 24,279,023 (11.1) |
| Non-Hispanic Asian | 13,923,299 (6.0) |
| Non-Hispanic Other | 9,931,477 (4.6) |
| Hispanic | 36,549,763 (16.8) |
| **Education level** |  |
| Less than HS | 13,877,108 (6.4) |
| 12 years or completed HS | 45,284,648 (20.8) |
| Some college | 85,345,020 (39.2) |
| College graduate or higher | 73,356,908 (33.7) |
| **Annual household income** |  |
| <20k | 29,756,093 (13.7) |
| 20-<35k | 23,442,753 (10.8) |
| 35-<50 | 25,677,118 (11.8) |
| 50-<75k | 40,507,802 (18.6) |
| 75k and more | 98,479,918 (45.2) |
| **Personal history of cancer** |  |
| Yes | 20,906,014 (9.6) |
| No | 196,957,670 (90.4) |
| **Family history of cancer** |  |
| Yes | 141,683,327 (65.0) |
| No | 76,180,357 (35.0) |
| Abbreviations: HINTS, Health Information Trends Survey; HS, high school; SD, standard deviation. | |
| ^a^Data was presented as weighted number (weighted %). | |
