# Supplementary material for: Trends in awareness and uptake of genetic testing among the United States population, 2011–2022
Source: J Community Genet. 2026 Aug 10;17(4):95. doi: 10.1007/s12687-026-00924-5 (PMC13457883; doi:10.1007/s12687-026-00924-5)
Supplement: Supplementary file 2 [file 12687_2026_924_MOESM2_ESM.docx]

**Table S4**. Weighted logistic regression models of genetic testing uptake overall, HINTS 2020-2022.

|  | **Genetic testing uptake** | |
| --- | --- | --- |
|  | **Weighted %** | **OR (95% CI)^a^** |
| **Number of participants** | 6511 |  |
| **Age** |  |  |
| 18-34 | 22.6 (19.1 to 26.5) | 1 [Reference] |
| 35-49 | 31.2 (27.7 to 34.9) | 1.45 (1.07-1.96) |
| 50-64 | 29.9 (27.1 to 32.8) | 1.31 (1.01-1.69) |
| 65-74 | 30.1 (26.7 to 33.8) | 1.24 (0.91-1.71) |
| 75+ | 35.4 (30.1 to 41.1) | 1.53 (1.05-2.22) |
| **Gender** |  |  |
| Female | 33.2 (31.1 to 35.4) | 1 [Reference] |
| Male | 23.5 (21.1 to 26.2) | 0.63 (0.53-0.76) |
| **Race/ethnicity** |  |  |
| Non-Hispanic White | 30.3 (28.2 to 32.6) | 1 [Reference] |
| Non-Hispanic Black | 24.0 (19.7 to 28.8) | 0.83 (0.63-1.09) |
| Non-Hispanic Asian | 20.2 (13.9 to 28.4) | 0.60 (0.38-0.96) |
| Non-Hispanic Other | 41.1 (30.7 to 52.4) | 1.69 (1.04-2.74) |
| Hispanic | 22.3 (18.6 to 26.4) | 0.80 (0.60-1.06) |
| **Education level** |  |  |
| Less than high school | 19.0 (11.1 to 30.7) | 0.79 (0.39-1.58) |
| 12 years or completed high school | 24.6 (20.0 to 29.8) | 0.94 (0.67-1.30) |
| Some college | 27.1 (23.9 to 30.7) | 1 [Reference] |
| College graduate or higher | 33.3 (31.0 to 35.7) | 1.23 (0.97-1.56) |
| **Annual household income** |  |  |
| <20k | 22.5 (18.1 to 27.7) | 0.68 (0.49-0.94) |
| 20-<35k | 22.8 (17.5 to 29.1) | 0.66 (0.45-0.96) |
| 35-<50 | 32.1 (26.3 to 38.5) | 1.04 (0.75-1.45) |
| 50-<75k | 23.5 (20.2 to 27.2) | 0.67 (0.53-0.84) |
| 75k and more | 32.1 (29.6 to 34.8) | 1 [Reference] |
| **Personal history of cancer** |  |  |
| No | 27.3 (25.5 to 29.3) | 1 [Reference] |
| Yes | 39.7 (34.7 to 45.0) | 1.44 (1.07-1.92) |
| **Family history of cancer** |  |  |
| No | 24.3 (21.0 to 27.9) | 1 [Reference] |
| Yes | 30.2 (28.2 to 32.2) | 1.17 (0.93-1.47) |
| ^a^Adjusted for age (18-34, 35-49, 50-64, 65-74, 75+), gender (male, female), race/ethnicity (non-Hispanic White, non-Hispanic Black, non-Hispanic Asian, non-Hispanic Other, Hispanic), education level (less than high school, 12 years or completed high school, some college, college graduate or more), annual household income (less than $20,000, $20,000 to <$35,000, $35,000 to <$50,000, $50,000 to <$75,000, $75,000 or more), personal history of cancer (yes, no), family history of cancer (yes, no), and cycle (HINTS 5 Cycle 4, HINTS 6). | | |
