# Supplementary material for: Trends in awareness and uptake of genetic testing among the United States population, 2011–2022
Source: J Community Genet. 2026 Aug 10;17(4):95. doi: 10.1007/s12687-026-00924-5 (PMC13457883; doi:10.1007/s12687-026-00924-5)
Supplement: Supplementary file 3 [file 12687_2026_924_MOESM3_ESM.docx]

**Table S2**. Sample size for crude weighted trends in genetic testing awareness and uptake among the US population, HINTS 2011-2022.

| **HINTS cycle years** | | | | | | |
| --- | --- | --- | --- | --- | --- | --- |
| **2011** | **2012** | **2013** | **2014** | **2017** | **2020** | **2022** |
| **Genetic testing awareness** | | | | | | |
| **N** | | | | | | |
| 3271 | 2806 | 2373 | 2875 | 2641 | 3104 | 5186 |
| **Weighted N** | | | | | | |
| 9,848,383,626 | 9,689,730,346 | 9,534,554,326 | 9,832,371,206 | 10,255,283,524 | 10,740,704,320 | 10,893,389,976 |
|  | | | | | | |
| **Genetic testing uptake** | | | | | | |
| **N** | | | | | | |
| - | - | - | - | - | 2841 | 4030 |
| **Weighted N** | | | | | | |
| - | - | - | - | - | 174,536,664 | 172,514,071 |
