# Supplementary material for: Trends in awareness and uptake of genetic testing among the United States population, 2011–2022
Source: J Community Genet. 2026 Aug 10;17(4):95. doi: 10.1007/s12687-026-00924-5 (PMC13457883; doi:10.1007/s12687-026-00924-5)
Supplement: Supplementary file 4 [file 12687_2026_924_MOESM4_ESM.docx]

**Table S3**. Weighted logistic regression models of health-related genetic testing awareness, HINTS 2020-2022.

|  | **Health-related genetic testing awareness** | |
| --- | --- | --- |
|  | **Weighted % (95% CI)** | **OR (95% CI)^a^** |
| **Number of participants** | 1889 |  |
| **Age** |  |  |
| 18-34 | 15.0 (9.1 to 23.8) | 1 [Reference] |
| 35-49 | 18.9 (13.6 to 25.8) | 1.27 (0.69-2.33) |
| 50-64 | 19.7 (15.3 to 25.0) | 1.40 (0.72-2.74) |
| 65-74 | 16.7 (12.4 to 22.1) | 1.17 (0.63-2.17) |
| 75+ | 12.3 (8.1 to 18.2) | 0.88 (0.46-1.68) |
| **Gender** |  |  |
| Female | 22.1 (18.3 to 26.5) | 1 [Reference] |
| Male | 12.6 (9.3 to 16.9) | 0.49 (0.33-0.72) |
| **Race/ethnicity** |  |  |
| Non-Hispanic White | 14.7 (11.0 to 19.3) | 1 [Reference] |
| Non-Hispanic Black | 26.1 (18.1 to 36.1) | 1.97 (0.99-3.91) |
| Non-Hispanic Asian | 15.1 (8.1 to 26.4) | 0.87 (0.45-1.69) |
| Non-Hispanic Other | 29.1 (10.7 to 58.5) | 2.33 (0.70-7.75) |
| Hispanic | 14.4 (10.8 to 19.1) | 1.13 (0.71-1.78) |
| **Education level** |  |  |
| Less than high school | 11.5 (7.0 to 18.5) | 0.70 (0.36-1.39) |
| 12 years or completed high school | 15.2 (11.4 to 20.1) | 0.91 (0.54-1.51) |
| Some college | 16.6 (12.2 to 22.2) | 1 [Reference] |
| College graduate or higher | 24.2 (17.7 to 32.1) | 1.60 (0.93-2.76) |
| **Annual household income** |  |  |
| <20k | 16.5 (11.7 to 22.9) | 0.77 (0.40-1.51) |
| 20-<35k | 13.6 (9.7 to 18.7) | 0.63 (0.38-1.06) |
| 35-<50k | 15.3 (9.8 to 23.2) | 0.78 (0.42-1.45) |
| 50-<75k | 16.5 (11.7 to 22.7) | 0.88 (0.43-1.79) |
| 75k and more | 21.0 (14.3 to 29.6) | 1 [Reference] |
| ^a^Adjusted for age (18-34, 35-49, 50-64, 65-74, 75+), gender (male, female), race/ethnicity (non-Hispanic White, non-Hispanic Black, non-Hispanic Asian, non-Hispanic Other, Hispanic), education level (less than high school, 12 years or completed high school, some college, college graduate or more), annual household income (less than $20,000, $20,000 to <$35,000, $35,000 to <$50,000, $50,000 to <$75,000, $75,000 or more), and cycle (HINTS 5 Cycle 4, HINTS 6). | | |
