# Supplementary material for: Trends in awareness and uptake of genetic testing among the United States population, 2011–2022
Source: J Community Genet. 2026 Aug 10;17(4):95. doi: 10.1007/s12687-026-00924-5 (PMC13457883; doi:10.1007/s12687-026-00924-5)
Supplement: Supplementary file 5 [file 12687_2026_924_MOESM5_ESM.docx]

**Figure S1.** Flow chart of the study population
